# Biocontrol agent of root-knot nematode *Meloidogyne* *javanica* and root-rot fungi, *Fusarium* *solani* in okra morphological, anatomical characteristics and productivity under greenhouse conditions

**DOI:** 10.1038/s41598-023-37837-z

**Published:** 2023-07-09

**Authors:** Waleed M. Ali, M. A. Abdel-Mageed, M. G. A. Hegazy, M. K. Abou-Shlell, Sadoun M. E. Sultan, Ehab A. A. Salama, Ahmed Fathy Yousef

**Affiliations:** 1 Department of Horticulture, College of Agriculture, University of Al-Azhar (Branch Assiut), Assiut, 71524 Egypt; 2 Agricultural Zoology and Nematology Department, Faculty of Agriculture, Al-Azhar University (Assiut Branch), Assiut, 71524 Egypt; 3 Department of Agricultural Botany (Plant Pathology), Faculty of Agriculture, Al-Azhar University (Assiut Branch), Assiut, 71524 Egypt; 4 Department of Agricultural Botany (General Botany), Faculty of Agriculture, Al-Azhar University (Assiut Branch), Assiut, 71524 Egypt; 5 Agricultural Botany Department, Faculty of Agriculture Saba Basha, Alexandria University, Alexandria, 21531 Egypt; 6 Department of Plant Biotechnology, Centre for Plant Molecular Biology and Biotechnology, TNAU, Coimbatore, 641003 India

**Keywords:** Cell biology, Microbiology, Plant sciences, Stem cells, Structural biology

## Abstract

This study was conducted to evaluate the ability of some fungal culture filtrate, as biocontrol agents against okra wilt caused by *Fusarium* *solani*. and *Meloidogyne* *javanica*. In the present study, fungal culture filtrates (FCFs) of *Aspergillus* *terreus* (1), *Aspergillus* *terreus* (2), *Penicillium* *chrysogenum,* and *Trichoderma* spp. were tested against *M.* *javanica* in vitro*.* The effects of *P.* *chrysogenum* and *Trichoderma* spp. (FCFs) in controlling root-rot fungi and root-knot nematode disease complex on okra plants were studied under greenhouse conditions (In vivo). In vitro experiment, the results revealed cumulative rate of J_2_s mortality of *M.* *javanica* reached to 97.67 and 95% by *P.* *chrysogenum* and *Trichoderma* spp., respectively, after 72 h. incubation. Additionally, *Trichoderma* spp exhibited the most effective inhibitory activity against the pathogen's radial growth, with a percentage of 68%. *P.* *chrysogenum* ranked second with 53.88%, while *A.* *terreus* (2) demonstrated the weakest inhibitory effect of 24.11%. T6 [Nematode infection (*M.* *javanica*) + Fungus infection (*F.* *solani*) + Overflowed with fungal culture filtrate (*P.* *chrysogenum*)] and T8 [Nematode infection (*M.* *javanica*) + Fungus infection (*F.* *solani*) + spray with fungal culture filtrate (*P.* *chrysogenum*)] had the greatest effects on nematode galling indices on okra roots and substantially reduced the reproductive factors in the greenhouse (In vivo experiment). T6 was the best treatment to decrease disease severity, as reached (28%) relatively. On the other hand, T12 [(Fungus infection (*F.* *solani*) + (Dovex 50% fungicide with irrigation water)] recorded the lowest disease severity reaching (8%) relatively. The results showed that nematode infection or fungus infection or both decreased all studied anatomical characteristics of okra root, stem, and leaves. We concluded from this study that root-knot nematode and root-rot fungi were reduced by using fungal culture filtrates and could improve plant growth.

## Introduction

Okra (*Abelmoschus* *esculentus* L.) Moench is a highly nutritious crop grown in regions with Mediterranean, tropical, and subtropical climates^1^. However, okra production is often hindered by a range of insect and disease problems, including root-knot nematodes (*Meloidogyne* spp.) and fungal pathogens^2^. These pests can significantly reduce crop yields, resulting in economic losses for farmers and food shortages for communities.

Plant-parasitic nematodes are among the most damaging pests of vegetable crops, accounting for significant global agricultural production losses each year^3^. Root-knot nematodes are the most common and commercially significant nematode pests, with a life cycle of 25 days at 27 °C^4^. Nematode and fungal infestations are considered the most important biotic factors that lower crop production, and nematodes can also predispose plants to secondary invasion by fungal diseases, exacerbating disease severity by modifying plant roots^5^.

Chemical control of nematodes and fungi is costly and has potential environmental and public health risks^6,7^. Therefore, developing safe and effective techniques for managing these harmful infections is essential. Biological control is a safer alternative to chemical nematicides, and *Penicillium* and *Trichoderma* fungi have a reputation for being hostile to plant-parasitic nematodes^8,9^. However, little is known about the mode of action of fungal culture filtrates (FCF) and their efficacy in controlling complex diseases caused by root-knot nematodes and rot-root fungi in okra plants.

In this study, we investigate the impact of biological control agents' strategy (fungal culture filtrates) in the control of the complex disease caused by the root-knot nematode and rot-root fungi. We also examine the mode of action of FCF on the morphological traits, anatomical analysis, and productivity of okra plants. Our study aims to contribute to the development of safe and effective techniques for the prevention and control of pathogenic agents in okra production.

## Materials and methods

### Isolation and identification of the causal pathogens

On Potato Dextrose Agar (PDA) medium, the causative pathogenic fungi were isolated using the agar plate technique^10^. Using the conventional hyphal tip approach, *Fusarium* spp. was isolated from the diseased Okra plants and introduced into pure culture^11^. To stop bacterial growth in the medium before sterilization, 250 mg L^−1^ of chloramphenicol (l-chloramphenicol) was added^12^. Utilizing either the single spore or hyphal tip procedures, the produced fungus were purified^13^**.** For subsequent studies, stock cultures were kept on PDA slants in a refrigerator at 5 °C. The physical characteristics of mycelia and spores, as described by Leslie and Summerell^14^ and Ismail, et al.^15^ were used to identify fungal isolates.

### Isolation and identification of endophytic fungi

The modified approach developed by Hallmann, et al.^16^ was used to isolate endophytic fungi. The obtained root and branch samples were carefully cleaned with sterile distilled water after being washed with mild detergent and running tap water to get rid of the dirt particles and adherent debris. For one minute, 70% ethanol was used to surface sterilize the samples. The samples were submerged in 4% sodium hypochlorite for 3 min to thoroughly sterilize the surfaces and get rid of any clinging germs. The samples were then dried on sterile filter paper after being washed with sterile distilled water. Using a sterile scalpel, the samples were divided into 5–10 mm pieces and grown in Petri plates with PDA media supplemented with chloramphenicol (250 mg L^–1^) to inhibit bacterial growth. Petri plates were covered with parafilm, incubated for 15 days at 27 ± 2 °C in the dark, and daily checked. Separate transfers of the purified endophytic fungal isolates to PDA slants were made. Finally, all the purified endophytic fungi were stored at 4 °C until further use. According to Domsch et al.^17^, Domsch et al.^18^, endophytic fungus isolates were recognized at the Assiut University Moubasher Mycological Center (AUMMC), Assiut, Egypt based on their macro- and microscopic features. Endophytic fungi *P.* *chrysogenum* and *Trichoderma* spp. were grown on potato dextrose broth (PDB) and incubated at 25 °C for 21 days, then filtered through sterilized cheesecloth to remove mycelium mats, then filtered through Membrane Solutions MS CA Syringe Filter (0.45 μm) to obtain cell-free culture filtrates then kept in freezer (at 5 °C) as stander solutions until use.

### Nematode inoculums

A pure culture of *M.* *javanica* was grown from a single egg mass removed from infected okra roots and kept alive on tomato cv. Strian-B in greenhouse. The whole root system of infected plants was dipped in water and carefully cleaned to eliminate any clinging dirt before being uprooted from the soil. Forceps were used to remove egg masses, which were subsequently cleaned with sterile water, put in a 0.5% sodium hypochlorite (NaOCl) solution, stirred for four minutes, and then dried on a 26 µm sieve^19^. A modified version of Baermann funnel technique was used to incubate the eggs for 3–5 days in order to produce second-stage juveniles (J_2_) for in vitro and pot studies^20^.

### In vitro experiment

#### Evaluation of fungal culture filtrates on *M. javanica* mortality

The assessment was done in 5 cm clean Petri dishes. As a control, Petri plates filled with distilled water were used. *A.* *terreus* (1), *A.* *terreus* (2), *P.* *chrysogenum*, and *Trichoderma* spp. fungal culture filtrates of concentration S/5 were prepared by adding 5 mL of distilled water to 1 mL of standard concentration "S" to study the juvenile’s mortality. One hundred from second stage (J_2_) of *M.* *javanica* were used in the study. Three replications of each treatment were used. All dead and alive (J_2_) were counted after 72 h of incubation to determine the proportion of J_2_ mortality.

#### Evaluation of fungal culture filtrates on *F. solani* radial colony growth

The study aimed to evaluate the effectiveness of culture filtrates derived from endophytic fungi in controlling *F.* *solani*. To achieve this, sterilized Petri dishes were filled with culture filtrates of *A.* *terreus* (1), *A.* *terreus* (2), *P.* *chrysogenum*, and *Trichoderma* spp., all at a 10% concentration (v/v). The PDA medium was allowed to solidify before placing 6 mm diameter mycelia discs of *F.* *solani* obtained from actively growing colonies in the center of the solidified agar plates. The control group was treated with sterilized distilled water instead of culture filtrate. The Petri dishes were then incubated at 25 ± 2 °C for four days, after which the percentage of inhibition in radial colony growth was calculated by comparing the results with the control group. The percent inhibition of mycelia growth of the pathogens was calculated using the following formula^21^.

$${\text{Inhibition }}\left( \% \right) \, = \, \left( {{\text{D1 }}{-}{\text{ D2}}} \right) \, /{\text{ D1 }} { \times }{ 1}00$$

where, D1 = colony diameter in control plate and D2 = colony diameter in treated plate.

### Greenhouse test

The plant experiments complied with local and national regulations and followed the rules of the Al-Azhar University Assiut branch (Assiut, Egypt). We have permission that our studies have complied with the relevant institutional, national, and international guidelines and legislation. The studies were conducted at the experimental greenhouse of Plant Pathology Department, Faculty of Agriculture, Al-Azhar University Assiut branch (27°12′16.67′′ N; 31°09′36.86′′ E). Okra seeds of the cultivar OH-102 were sterilized for 3 min with 1% sodium hypochlorite, then gently rinsed with sterilized distilled water, followed by thorough drying. Silt and clay soil that had been formalin-disinfested were mixed in a ratio of one to one in 25 cm diameter plastic pots. On autoclaved sand barley medium, inocula of *F.* *solani* were produced by cultivating the required causative pathogenic fungus at 25 °C for 2 weeks. The prepared inoculum was mixed thoroughly with the potted soil before plantingat a rate of 3% weight and left for a week. As a control, potted soil was combined at the same rate with pathogen-free sterilized sand barley. Okra seeds that had been surface sterilized and appeared to be in good condition were sown in pots at a rate of four seeds per pot. Each treatment utilized five replicated pots. The okra plants were injected with 3000 freshly hatched juveniles derived from the pure culture of *M.* *javanica* two weeks after planting. The necessary quantity of nematode suspension was injected into three holes drilled around the plants to perform the inoculation. To keep the holes from drying out, the soil was placed over them.

### Experimental design and treatments

The experiment was carried out in a randomized complete block design. The treatments for the experiment were:

T1 [Nematode infection (*M.* *javanica*)].

T2 [Nematode infection (*M.* *javanica*) + Overflowed with fungal culture filtrate (*P.* *chrysogenum*)].

T3 [Nematode infection (*M.* *javanica*) + Overflowed with fungal culture filtrate (*Trichoderma* spp.)].

T4 [Nematode infection (*M.* *javanica*) + (Oxamyl 24% pesticide with irrigation water)].

T5 [Nematode infection (*M.* *javanica*) + Fungus infection (*F.* *solani*)].

T6 [Nematode infection (*M.* *javanica*) + Fungus infection (*F.* *solani*) + Overflowed with fungal culture filtrate (*P.* *chrysogenum*)].

T7 [Nematode infection (*M.* *javanica*) + Fungus infection (*F.* *solani*) + Overflowed with fungal culture filtrate (*Trichoderma* spp.)].

T8 [Nematode infection (*M.* *javanica*) + Fungus infection (*F.* *solani*) + spray with fungal culture filtrate (*P.* *chrysogenum*)].

T9 [Nematode infection (*M.* *javanica*) + Fungus infection (*F.* *solani*) + spray with fungal culture filtrate (*Trichoderma* spp.)].

T10 [Fungus infection (*F.* *solani*) + Overflowed with fungal culture filtrate (*Trichoderma* spp.)].

T11 [Fungus infection (*F.* *solani*) + Overflowed with fungal culture filtrate (*P.* *chrysogenum*)].

T12 [(Fungus infection (*F.* *solani*) + (Dovex 50% fungicide with irrigation water)].

T13 Fungus infection (*F.* *solani*).

T14 [Control (untreated)].

Fungal culture filtrates (FCFs) were applied to plants as overflowed or sprayed onto leaf surface four days after inoculation by nematode (two weeks after planting). Oxamyl 24% and Dovex 50% were applied in the dose recommended by the manufacturer for resistance to nematodes and Fungus, respectively.

During the experiment, crop measurements (the number of fruits, fruit length, and fruit weight) every 3 days were estimated. After 8 weeks after inoculation, the plants were carefully uprooted from the soil and measured for shoot length, root length, plant weight, stem diameter, number of branches, leaf area, and leaf number. Additionally, the number of galls, egg-masses/root, and eggs/egg-mass were counted. The ultimate nematode population and nematode reproduction rate were estimated.

According to Abdel-Razik, et al.^22^, the Fusarium wilt disease severity (%) was determined after 60 days had passed since planting using the disease severity (DS) scale (0:5), which indicated [0 = no visible symptoms; 1 = slight vein-clearing and chlorosis of the leaves; 2 = yellowing and wilting of lower leaves and extend to upper leaves; 3 = brown (discoloration) of the vascular systems of tap rot and stem; 4 = indicates necrotic streaks on the stem base that extend toward the apex, while 5 = indicates an early plant demise. The following equation was used to calculate the percentage of disease severity index (DSI) for each tested isolate:

$${\text{DSI }}\% \, = \, (\Sigma {\text{d}}/{\text{d max }} { \times }{\text{ n}}) \, { \times }{ 1}00$$

where (d) is the overall disease rating for each plant, (d max) is the maximum disease rating, (n) is the total number of plants tested in each duplicate.

### Anatomical study

At 60 days following planting, a comparative anatomical investigation of the root, stem, and leaf of treated and control okra plants was conducted. Okra root, stem, and terminal leaf samples were gathered. The samples of stem and leaf were obtained from the 5th apical internode of the main stem and its corresponding leaf with the most successful treatments, i.e., T1, T5, T6, T7, and T13, in addition to those of the control (T14) after 60 days. The root tips were sampled at a distance of 0.5 cm. The samples were collected, killed, and preserved in a solution containing (5 mL formalin, 10 mL glacial acetic acid, and 85 mL 70% ethanol). It was then washed in 50% ethyl alcohol, dehydrated in a succession of ethyl alcohols (50, 70, 80, 90, 95, and 100%), infiltrated in xylene, embedded in paraffin wax with a melting temperature of 40–45 °C, sectioned on a rotary microtome at a thickness of 5–7 m, dyed with the double stain procedure (light green and safranin), cleared in xylene and mounted in Canada balsam^23^. Sections were examined to look for histological evidence of treatment-related noticeable responses. The prepared sections were examined under a microscope and counts and measurements (µ) were taken using computerized morphometrical analysis. This analysis was carried out using a Research Microscope type Axiostar plus manufactured by Zeiss that was upgraded to be able to use a professional digital image analysis system (Carl Zeiss Axiovision Product Suite DVD 30).

The following measurements were recorded: 1—Anatomical features of okra root: root diameter (Ø of root); cortex thickness; diameter of vascular cylinder (Ø of V.C); length of xylem arch. 2—Anatomical features of okra stem: stem diameter (Ø of stem); cortex thickness; diameter of vascular cylinder (Ø of V.C); vascular bundle thickness (V.B. thickness); pith thickness. 3—Anatomical features of okra leaf: thickness of leaf midrib; spongy tissue thickness; palisade tissue thickness; vascular bundle thickness (V.B. thickness).

### Statistical analysis

Three to four duplicate experiments were run for each treatment to satisfy the random design. One-way analysis of variance (ANOVA), which was performed using the software (Statistix 8.1) for statistical analysis of the experiment's results, was used to analyze the growth parameters and yield and assess for significance. To look at the variations between the means that were statistically significant, Duncan's multiple range tests with 95% confidence were performed^24^.

### Statement permission

The authors declare that they have proper permission.

## Results

### In vitro experiment

#### Effect of FCFs on root-knot nematode

**Figure 1:**
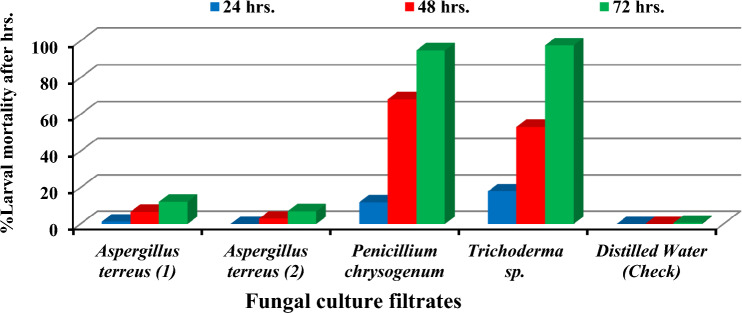
Effect of four fungal culture filtrates (FCFs) on mortality percentage of *M.* *javanica* J_2_ under laboratory conditions.

The fungal culture filtrates (FCFs) of *A.* *terreus* (1), *A.* *terreus* (2), *P.* *chrysogenum* and *Trichoderma* spp. were tested for their nematicidal effect on second stage juveniles of *M.* *javanica* in vitro (Fig. 1). The results revealed that the nematode mortalities with fungal culture filtrates were increased with the increase of the exposure time from 24 to 72 h (FCF) of *Trichoderma* spp. was more effective against *M.* *javanica* J_2_ followed by *P.* *chrysogenum* with mortality rates (97.67 & 95%), respectively, after 72 h of exposure time.

#### Effect of FCFs on *F. solani* radial colony growth

**Figure 2:**
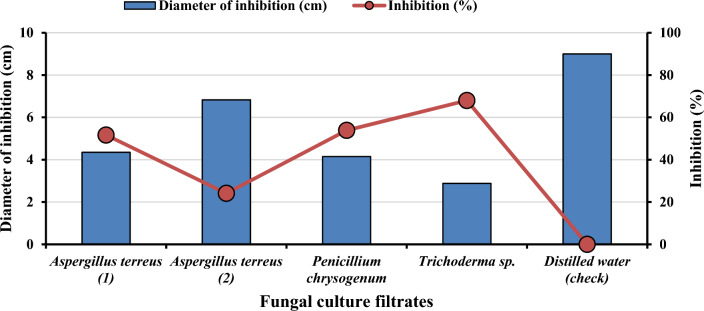
Effect of four fungal culture filtrates (FCFs) on *F.* *solani* radial growth under laboratory conditions after four days.

**Figure 3:**
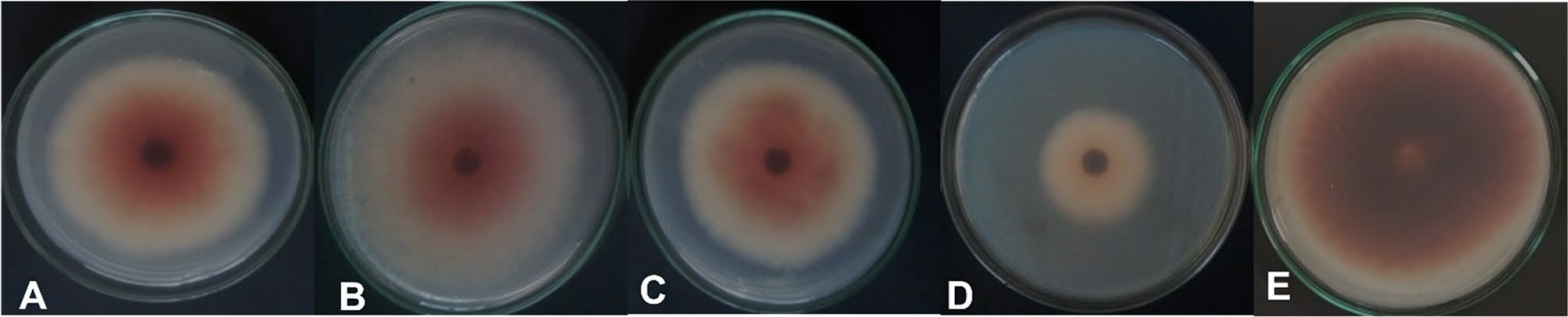
Antifungal activity of endophytic fungi culture filtrates against the causal pathogen *F.* *solani*. Where: (**A**) *A.* *terreus* (1); (**B**) *A.* *terreus* (2); (**C**) *P.* *chrysogenum*; (**D**) *Trichoderma* spp.; and (**E**) control.

An experiment was conducted in a laboratory to investigate the efficacy of fungal culture filtrates (FCFs) derived from endophytic fungi at a 10% concentration (v/v) as biocontrol agents against *F.* *solani*. The study involved testing the FCFs of *A.* *terreus* (1) and *A.* *terreus* (2), *P.* *chrysogenum*, and *Trichoderma* spp. against the causal pathogen (as shown in Figs. 2 and 3A–E). The results revealed that the FCFs inhibited the growth of the pathogen with varying degrees of effectiveness. Treatment with FCFs of these antagonistic fungi significantly reduced the linear growth of the tested pathogen compared to the control (distilled water). Notably, *Trichoderma* spp (Fig. 3D) showed the highest percent inhibition of radial growth of the causal pathogen (68%), followed by *P.* *chrysogenum* (53.88%) (Fig. 3C). In contrast, *A.* *terreus* (2) had the lowest inhibitory effect (24.11%) (Fig. 3B).

### Greenhouse experiment

#### Effect of fungal culture filtrates (FCFs) on root-rot fungus and root-knot nematode under greenhouse conditions

**Figure 4:**
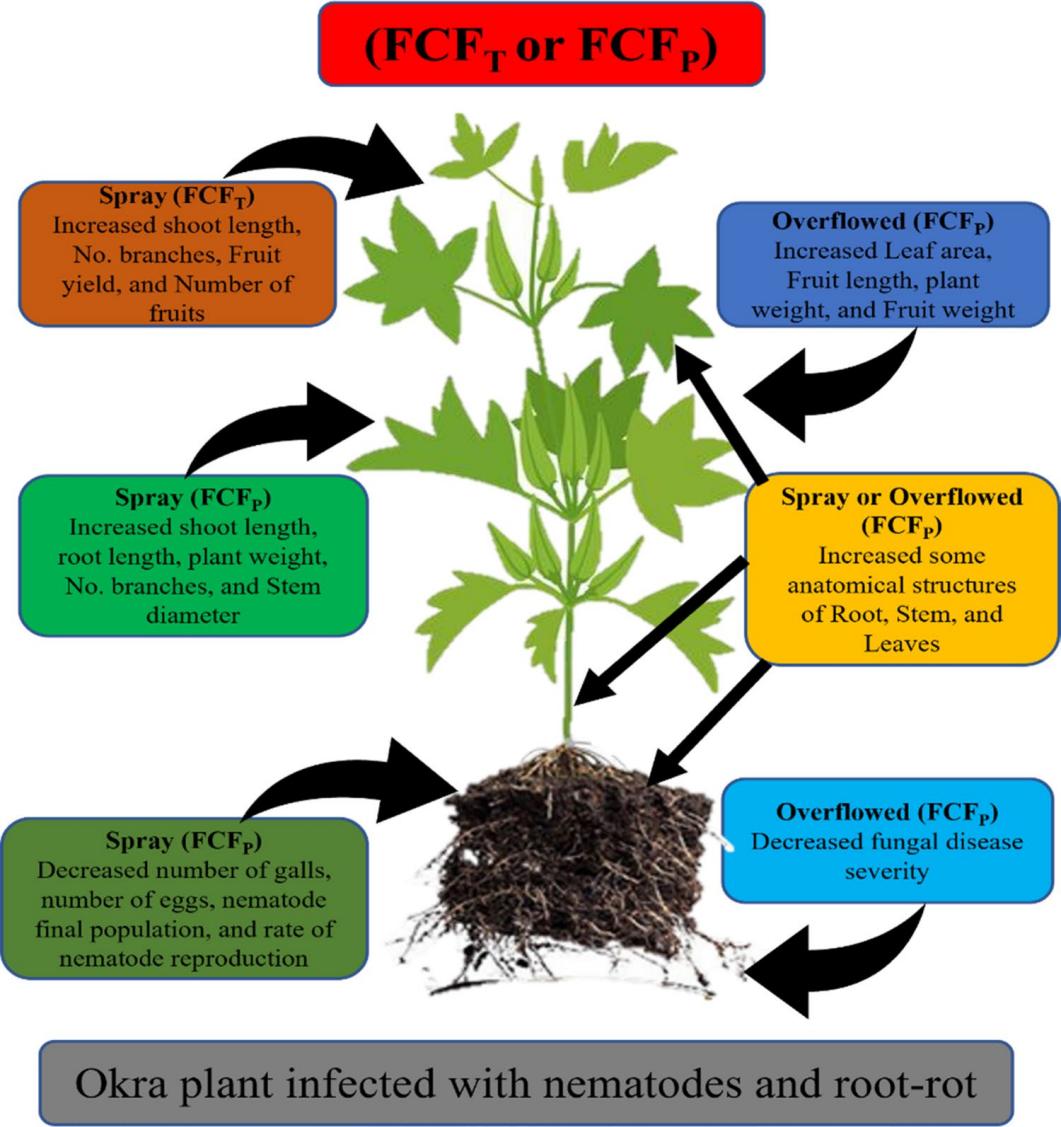
An illustration of the effect of spray or overflowed of *P.* *chrysogenum* (FCF_P_) and *Trichoderma* spp. (FCF_T_) infected with root-knot nematode or/and root-rot fungi infecting okra under greenhouses condition.

The effects of *P.* *chrysogenum* (FCF_P_) and *Trichoderma* spp. (FCF_T_) applied as foliar spray and soil drenches on control of root-rot fungus (*F.* *solani*) and root-knot nematode (*M.* *javanica*) disease complex on growth and anatomical characteristics of okra plants cv. OH-102 were studied under greenhouse conditions. Data presented in Table 1 and Fig. 4 showed that, all applications of the tested (FCFs) significantly reduced the values of root galls per root and nematode reproduction on roots of okra when compared with untreated plants [control (T14). Therefore, (FCF_P_) as soil drenches (T4) and foliar sprays (T8) caused the lowest gall numbers (40 and 49)], respectively. While (FCF_P_) as soil drenches (T4) and foliar sprays (T8) caused the lowest rate of nematode reproductions (2.32 and 4.42), respectively. On the other hand, plants inoculated with T1 recorded the highest number of galls and rates of nematode reproductions (170 and 21.20, respectively) followed by T5 (143 and 16.75, respectively). The highest values of the final nematode population and number of galls were under T1, while the lowest values of the final nematode population and number of galls were under T4.

**Table 1:** Effect of spray or overflowed of *P.* *chrysogenum* (FCF_P_) and *Trichoderma* spp. (FCF_T_) against root-knot nematode or/and root-rot fungi infecting okra under greenhouses condition.

Treatments	No. galls	Nematode population			Fungal disease severity (%)
		No. of eggs/egg-mass/root system	Nematode final population (P_f_)	Rate of nematode reproduction (P_f_/P_I_)	
T1	170a	411a	63,611	21.20	48
T2	50def	298b	14,243	4.75	36
T3	76c	323b	23,518	7.84	40
T4	40g	174c	6969	2.32	36
T5	143b	365eb	50,248	16.75	60
T6	54def	311b	16,743	5.58	28
T7	68cd	308b	20,378	6.79	36
T8	49fg	277b	13,255	4.42	36
T9	63cde	301b	18,673	6.22	40
T10	–	–	–	–	32
T11	–	–	–	–	48
T12	–	–	–	–	8
T13	–	–	–	–	36
T14	–	–	–	–	00

Means in each column followed by the same letters are not significantly different by (*p* = 0.05) according to Duncan's multiple range test. For more details about the treatments see experimental design and treatments section. Where rate of nematode reproduction = final population (P_f_)/initial population (P_I_); final population (P_f_) = larva in soil + developmental stages + adult female + (number of egg-masses × eggs); initial population (P_I_) was 3000 s stage larva (J_2_) of root knot nematode (*M.* *javanica*).

Data presented in Table 1 indicate that *F.* *solani* was able to infect okra plants causing root rot with varied degrees. Therefore, T5 caused the highest disease severity (60%) followed by T1 and T11 (48%). While the disease severity of T3 and T9 observed the same disease severity (40%) relatively. On the other hand, T6 were recorded the lowest disease severity as reached (28%) relatively followed by T12 (8%).

#### Morphological characteristics

Plant metrics were offered lowest significant, under the circumstances of single nematode infection, single fungal infection, or both of them together (Table 2). Generally, application of the fungal culture filtrates (FCF_P, T_) resulted in a significant increase in most of the measured traits, whether it was a spraying system or overflowed, the tallest shoot recorded by T12 and T8 were (42.71 and 42.64 cm), respectively, while the shortest shoot length observed under T5 with (21.51 cm). The characteristic of root length was significantly higher by spraying (FCF_T, P_) at treat nematode and fungal infection under T8, T9, and T12 were (34.81, 35.82, and 35.94 cm), respectively, and the lowest significant value noted at nematode and fungal infection T5 (19.1 cm). The biggest plant weight watched under T12 and T11 (49 and 48.33 g.) as well as the smallest weight mentioned was 16.67 g. with nematode and fungal infection (T5). Several significant were recognized in stem diameter, and the highest rang listed by (FCF_P_) applied as a spray on treatment nematode and fungal infection (T8) was 1.43 mm, whereas the less rang was 0.76 mm for nematode infection (T1). Branches number trait presented various significance, T4, T8, and T9 led to the highest number of branches (5.00, 4.33, and 4.33), respectively, however, the lowest number of branches noted at treat nematode and fungal infection (T5) was (2.00). Although the treatment T12 (Fungicide) was the highest treatment that recorded the highest leaves area (40.69 cm^2^), there was no significant difference between it and the application of the (FCF_P_) as overflowed for nematode and fungal infection (T6) and (FCF_T_) as a foliar spray (T9) where were 39.67 and 37.90 cm^2^, respectively, and the minimum leaves area was 16.41 cm^2^ by fungal infection (T1). Although the treatment T12 (Fungicide) was the highest treatment that recorded the highest leaves number (19.33), there was no significant difference between it and the application of the (FCF_P_) as overflowed to treat fungal alone (T11) and nematode alone (T2) had greater leaves number were 16.67 and 15.67, respectively, while the lowest leaves number was 3.67 under T1. Else, there were no significant differences between T12 [(Fungus infection (*F.* *solani*) + (Dovex 50% fungicide with irrigation water)] and T9 [Nematode infection (*M.* *javanica*) + Fungus infection (*F.* *solani*) + spray with fungal culture filtrate (*Trichoderma* spp.)] in the yield of fruits, as the yield of fruits was 34.77 g plant^−1^ under T12 and under T9 was 33.27 g plant^−1^.

**Table 2:** Effect of spray or overflowed of *P.* *chrysogenum* (FCF_P_) and *Trichoderma* spp. (FCF_T_) on plant growth parameters of okra plant under greenhouse conditions infected with root-knot nematode or/and root-rot fungi infecting.

Treatments	Shoot length (cm)	Root length (cm)	Plant weight (g)	Stem diameter (mm)	No. branches	Leaves area (cm^2^)	No. of leaves	Fruit yield (g) plant^−1^	No fruits plant^−1^	Fruit length (cm)	Fruit weight (g)
T1	24.87def	24.97ab	21cd	0.76d	2.33cd	16.41ef	3.67f	31.83abc	3.00b	9.37ab	9.50abc
T2	31.63b-e	26.10ab	40ab	1.15abc	3.67a-d	25.52cde	15.67ab	21.47ef	3.00b	9.30ab	9.20bcd
T3	26.66cdef	25.46ab	21.67cd	0.92cd	2.33cd	26.18cde	8.33def	24.23def	3.00b	8.73ab	8.07cde
T4	33.59a-d	27.87ab	42.33ab	1.13abc	5.00a	29.42bcd	15.33ab	30.03a-d	3.00b	9.93a	10.03ab
T5	21.51f	19.16b	16.67d	0.90cd	2.00d	12.72f	6.67ef	27.63b-e	3.00b	7.93b	7.17e
T6	32.56a-e	26.41ab	34.67abc	1.14abc	4.00abc	39.67ab	12.00bcd	32.37abc	3.00b	9.77a	11.13a
T7	31.32b-f	29.19ab	33.33bc	1.11abc	4.00abc	32.71abc	10.33cde	27.67b-e	3.00b	8.93ab	9.23bcd
T8	42.64a	34.81a	42.67ab	1.43a	4.33ab	34.11abc	14.00bc	25.83c-f	3.00b	9.30ab	9.67abc
T9	36.63abc	35.82a	43.00ab	1.05bcd	4.33ab	37.90ab	14.00bc	33.27ab	4.00a	9.53ab	10.43ab
T10	22.74ef	27.79ab	30.00bcd	0.96cd	2.33cd	18.51def	9.33cde	23.03ef	3.00b	8.50ab	10.00ab
T11	38.86ab	28.91ab	48.33a	1.17abc	3.67a-d	28.49bcd	16.67ab	32.50abc	3.33ab	9.60a	7.67de
T12	42.71a	35.94a	49.00a	1.31ab	3.00bcd	40.69a	19.33a	34.77a	3.00b	9.40ab	7.80 de
T13	32.43a-e	26.44ab	29.67bcd	1.08bcd	2.33cd	23.46cde	6.67ef	19.97f	3.00b	8.43ab	7.50 de
T14	33.55a-d	28.48ab	35abc	1.09bc	2.67bcd	33.32abc	7.00ef	23.40ef	4.00a	8.87ab	7.80 de

Means in each column followed by the same letters are not significantly different by (*p* = 0.05) according to Duncan's multiple range test. For more details about the treatments see experimental design and treatments section.

In this study, the (FCF_T_) as a foliar spray for nematode and fungal infection combined (T9) equal in the number of fruits with control treatment (T14). The greatest increase of fruit length was under T4 (9.93 cm) followed by (FCF_P_) as overflowed with treated nematode and fungal infection together and fungal infection T6 (9.77 cm), as well the lowest value was reported by nematode and fungal infection treatment T5 (7.93 cm). Application of the (FCF_P_) as overflowed with nematode and fungal infection combined (T6) achieved the weight of the fruits (11.13 g), followed by (FCF_T_) treat as a foliar spray with nematode and fungal infection combined T9 (10.43 g), while the lowest values noted by nematode and fungal infection treatment T5 (7.17 g).

#### Anatomical studies

The Anatomical studies to follow up the internal structure changes, which exhibited the most noticeable response against root-knot nematode, root-rot fungi, and fungal culture filtrate of *P.* *chrysogenum* applied as overflowed or foliar spray and their effect on the mean counts and measurements in micron (μ) of certain histological features of root, stem and leaf of okra grown in the plastic pots at 60 days after sowing based on transverse sections.

**Figure 5:**
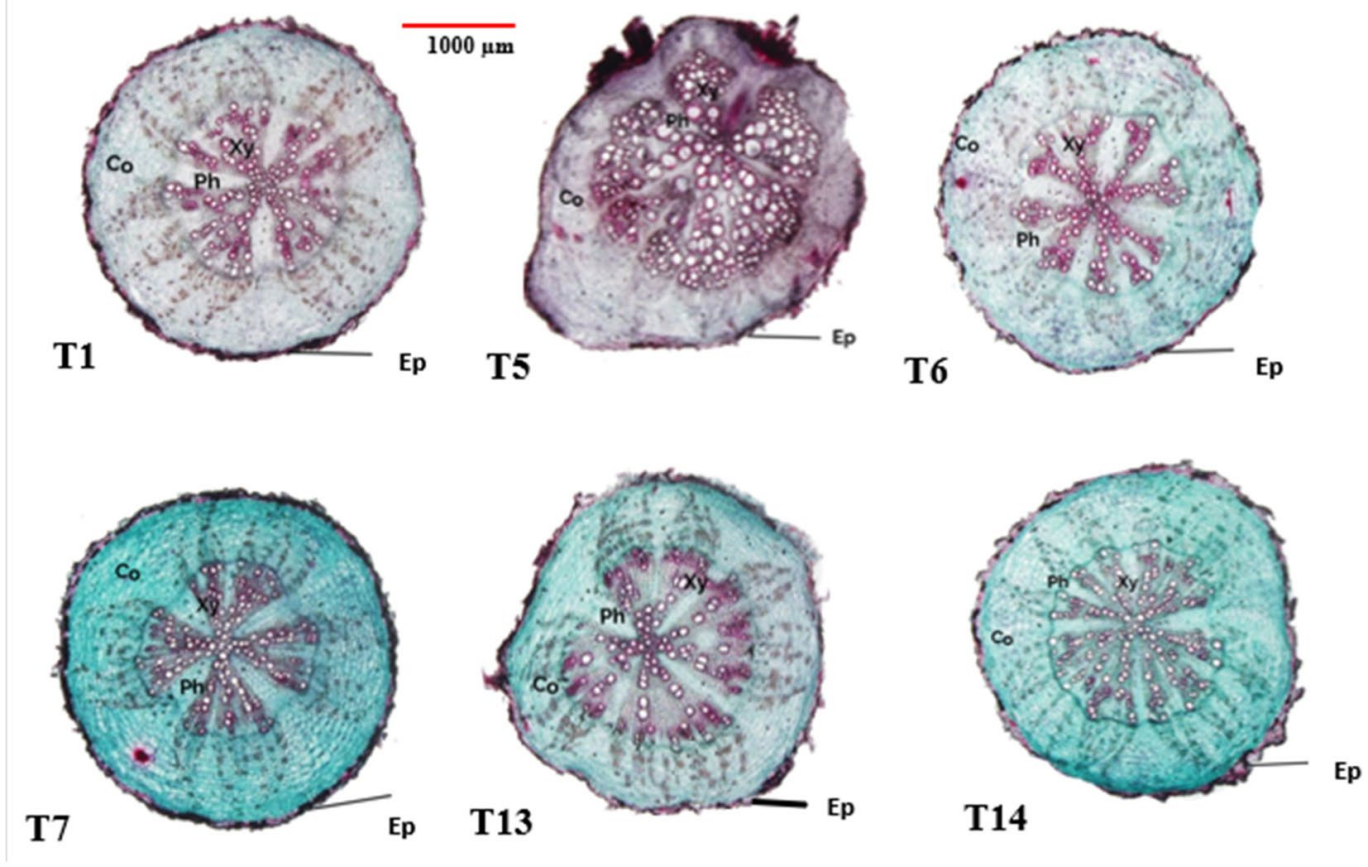
Transverse sections (×12.5) of okra roots at 60 days after sowing as affected by different applied treatments. Where: T1 = [Nematode infection (*M.* *javanica*)]; T5 = [Nematode infection (*M.* *javanica*) + Fungus infection (*F.* *solani*)]; T6 = [Nematode infection (*M.* *javanica*) + Fungus infection (*F.* *solani*) + Overflowed with fungal culture filtrate (*P.* *chrysogenum*)]; T7 = [Nematode infection (*M.* *javanica*) + Fungus infection (*F.* *solani*) + Overflowed with fungal culture filtrate (*Trichoderma* spp.)]; T13 = Fungus infection (*F.* *solani*); T14 = [Control (untreated)]; *Ep* epidermis, *Co* cortex tissue, *Ph* phloem tissue, *Xy* xylem tissue.

Data in Table 3 and Figs. 3, 4 and 5 obviously indicate the effect of different applied treatments: T1 [Nematode infection (*M.* *javanica*)], T5 [Nematode infection (*M.* *javanica*) + Fungus infection (*F.* *solani*)], T6 [Nematode infection (*M.* *javanica*) + Fungus infection (*F.* *solani*) + Overflowed with fungal culture filtrate (*P.* *chrysogenum*)], T7 [Nematode infection (*M.* *javanica*) + Fungus infection (*F.* *solani*) + Overflowed with fungal culture filtrate (*Trichoderma* spp.)], T13 [Fungus infection (*F.* *solani*)], and on different anatomical features of okra root, stem and leaves compared with untreated plants (T14). In current study, most of these applied treatments have a positively impact on most studied histological characteristics of different okra organs i.e., root (Ø of root, cortex thickness, Ø of V.C and length of xylem arch), for stem (Ø of stem, cortex thickness, Ø of V.C and V.B thickness) as well as leaves (spongy and palisade tissues thickness and V.B thickness).

**Table 3:** Anatomical characteristics of *P.* *chrysogenum* fungal culture filtrate (FCF_P_) against *M.* *javanica* and *F.* *solani* on okra root, stem and leaf under greenhouse conditions.

Treatment	Root anatomical			Stem anatomical			Leaves anatomical		
	Ø of root (µm)	Ø of V.C (µm)	Xylem arm length (µm)	Ø of stem (µm)	Ø of V.C (µm)	V.B. thickness (µm)	Palsied tissue thickness (µm)	spongy tissue thickness (µm)	V.B. thickness (µm)
T1	2951.79c	1621.39c	801.67bc	4317.11c	3073.24d	445.48c	143.51d	133.29c	193.14d
T5	2790.21d	1558.87d	741.27c	3807.31e	2807.99f	284.23e	141.79d	127.85c	170.67f
T6	3112.5b	1674.43b	804.16c	4325.69c	3211.79c	486.01b	167.08c	145.71b	205.11c
T7	3116.71b	1702.66b	837.38b	4474.02b	3346.29b	489.67a	193.41b	163.26a	224.96b
T13	2920.22c	1620.12c	773.48bc	3968.18d	2917.05c	334.07d	142.64d	128.53c	185.81e
T14	3400.54a	2163.78a	1276.26a	4625.94a	3532.19a	510.81a	201.89a	169.79a	271.79a
L.S.D. 5%	45.66	43.90	79.73	51.03	37.47	11.20	7.60	11.87	6.46

Where: T1 = [Nematode infection (*M.* *javanica*)]; T5 = [Nematode infection (*M.* *javanica*) + Fungus infection (*F.* *solani*)]; T6 = [Nematode infection (*M.* *javanica*) + Fungus infection (*F.* *solani*) + Overflowed with fungal culture filtrate (*P.* *chrysogenum*)]; T7 = [Nematode infection (*M.* *javanica*) + Fungus infection (*F.* *solani*) + Overflowed with fungal culture filtrate (*Trichoderma* *spp.*)]; T13 = Fungus infection (*F.* *solani*); T14 = [Control (untreated)].

The obtained results clearly showed that the application of T7 [Nematode infection (*M.* *javanica*) + Fungus infection (*F.* *solani*) + Overflowed with fungal culture filtrate (*Trichoderma* spp.)] and T6 [Nematode infection (*M.* *javanica*) + Fungus infection (*F.* *solani*) + Overflowed with fungal culture filtrate (*P.* *chrysogenum*)] treatments recorded the highest values of studied anatomical characteristics after untreated treatment (T14) compared with other applied treatments.

##### Root anatomy

In the present investigation, the treatments for nematode infection (T1), fungi infection (T13), or nematode infection + fungus infection (T5) lowered the morphological characteristics of the okra root most when compared to the control (T14), T6, and T7. The obtained findings are shown in Table 3 and Fig. 5. Numerous anatomical characteristics, particularly the T5, saw obvious reductions in their thickness. Since it had the most adverse impact on the anatomical traits that had been evaluated. The most significant anatomical characteristics were i.e., Ø of root (µm), Ø of V.C(µm), and Xylem arm length (µm), where all of them were significantly reduced with T5 compared with control (T14) and T7.

It's interesting to notice that the treatments' good effects on many anatomical characteristics were entirely undone when the treated plants' vegetative and reproductive development was increased, particularly in the cases of the control (T14) and (T7) treatments when compared to other treatments. The current investigation demonstrated increases in both xylem tissue, which is responsible for transporting various assimilates from leaves to fruits and other plant sinks, and phloem tissue, which is responsible for transporting water and mineral nutrients from roots to leaves. Therefore, the increase in the ultimate fruit yield might be directly attributed to the improvement of translocation events.

##### Stem anatomy

**Figure 6:**
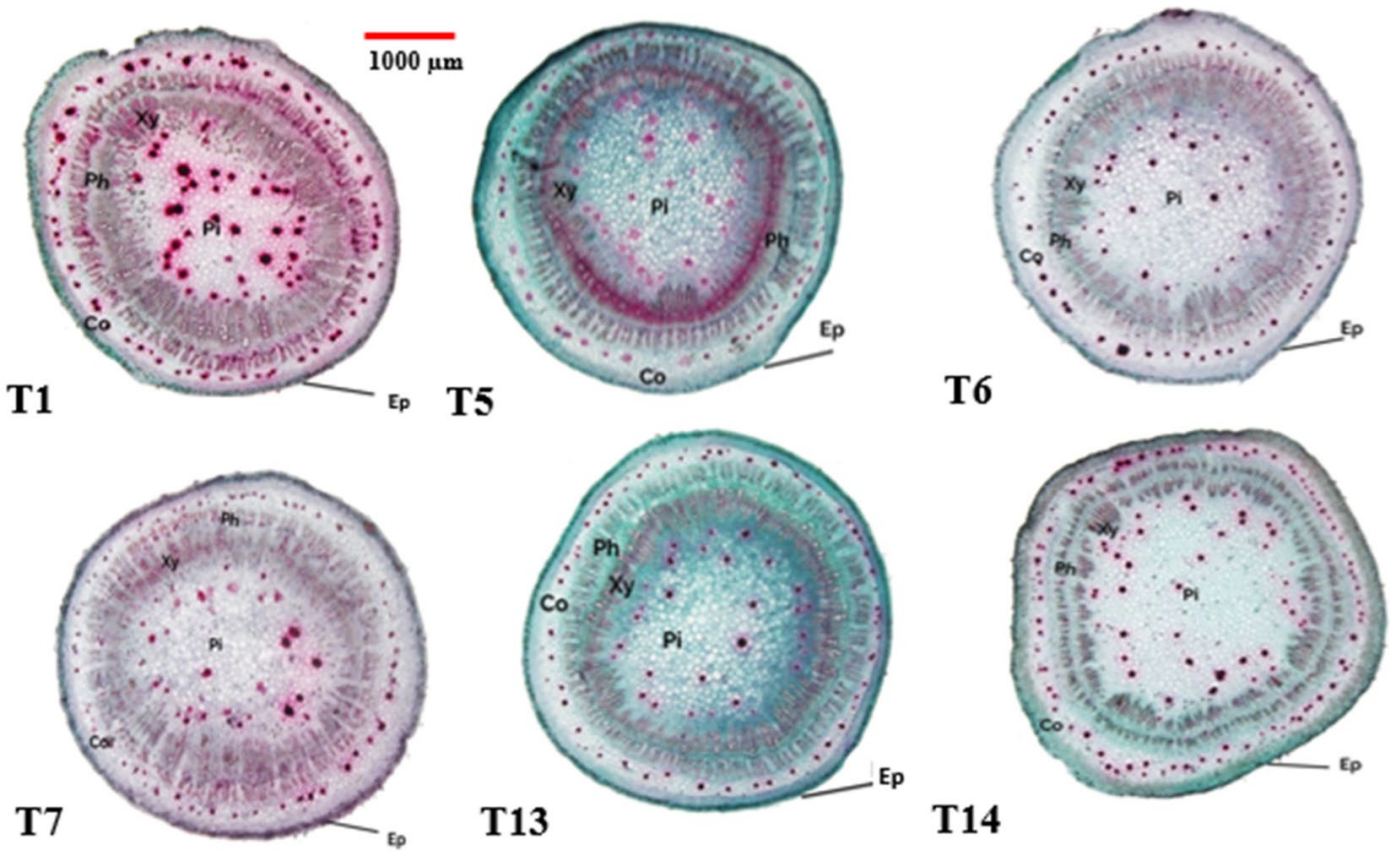
Transverse sections (×12.5) of okra stems at 60 days after sowing as affected by different applied treatments. Where: T1 = [Nematode infection (*M.* *javanica*)]; T5 = [Nematode infection (*M.* *javanica*) + Fungus infection (*F.* *solani*)]; T6 = [Nematode infection (*M.* *javanica*) + Fungus infection (*F.* *solani*) + Overflowed with fungal culture filtrate (*P.* *chrysogenum*)]; T7 = [Nematode infection (*M.* *javanica*) + Fungus infection (*F.* *solani*) + Overflowed with fungal culture filtrate (*Trichoderma* spp.)]; T13 = Fungus infection (*F.* *solani*); T14 = [Control (untreated)]; *Ep* epidermis, *Co* cortex tissue, *Ph* phloem tissue, *Xy* xylem tissue, *Pi* pith tissue.

The obtained results in Table 3 and Fig. 6 show that, the treatments of nematode infection (T1) or fungi infection (T13) or nematode infection + fungus infection (T5) in the present study decreased the most of okra stem anatomical features compared with the (T14), T6, and T7. Okra plants with control (T14) and T7 appeared to be the most effective treatment. This treatment manifested the best results as it exceeded that of nematode infection + fungus infection (T5) in terms of most studied anatomical features. With regard to the stem diameter, it was reached its maximum value (3807.31 eμ) with nematode infection + fungus infection (T5), comparing with other treatments. Application treatments of control (T14) and T7 recorded highly values of the stem studied anatomical characteristics especially, in the Ø of root (µm), Ø of V.C (µm)and xylem arm length (µm) compared with other applied treatments.

It's interesting to notice that improving the vegetative and reproductive development of treated plants fully reversed these beneficial responses of various anatomical characteristics to treatments, particularly in the case of control (T14) and T7 compared to other treatments. The current investigation therefore demonstrated such expansions of xylem tissue, or the conduit of various assimilates from leaves to fruits and other plant sinks, and phloem tissue, or the route of water and mineral nutrient translocation from roots to leaves. As a result, an increase in the ultimate fruit production might be directly attributed to better translocation occurrences.

##### Leaf anatomy

**Figure 7:**
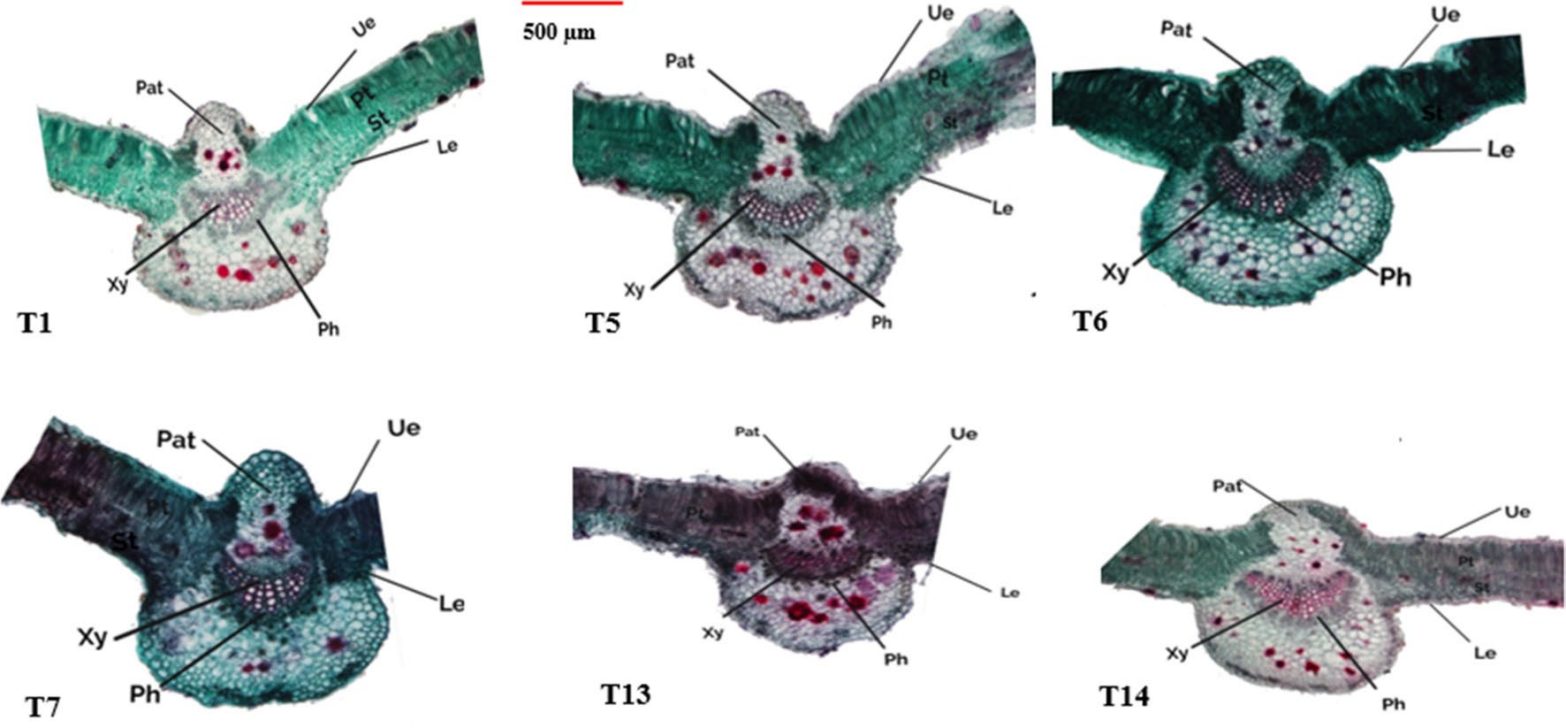
Transverse sections (×100) of Okra leaves at 60 days after sowing as affected by different applied treatments. Where: T1 = [Nematode infection (*M.* *javanica*)]; T5 = [Nematode infection (*M.* *javanica*) + Fungus infection (*F.* *solani*)]; T6 = [Nematode infection (*M.* *javanica*) + Fungus infection (*F.* *solani*) + Overflowed with fungal culture filtrate (*P.* *chrysogenum*)]; T7 = [Nematode infection (*M.* *javanica*) + Fungus infection (*F.* *solani*) + Overflowed with fungal culture filtrate (*Trichoderma* spp.)]; T13 = Fungus infection (*F.* *solani*); T14 = [Control (untreated)]; *Ue* upper epidermis, *le* lower epidermis, *ph* phloem tissue, *xy* xylem tissue.

The information in Table 3 and Fig. 7 demonstrated how the examined histological characteristics of the leaf reacted similarly to the anatomical characteristics of the stem. For mesophyll tissue, the application of *M.* *javanica* (T1), *F.* *solani* (T13), and *M.* *javanica* + *F.* *solani* had a significant impact on the thickness of both the spongy and palisade tissues (T5). Here, spongy tissue thickness value was decreased (127.85 cμ) with T5 but increased to reach (133.29 cμ) and (128.53 cμ) with T1 and T13, respectively, which were the more effective treatments in the same order. By contrast, palisade tissue thickness was (143.51 d and 142.64 dμ) with T1 and T13, respectively, but decreased to reach (141.79 dμ) with T5. With regard to V.B. thickness (µm) was reached their maximum values with T5 treatment. Here, V.B. thickness (µm) was decreased (170.67 fμ) with T5 while increased to reach (193.14 d) and (185.81 eμ) with T1 and T13, respectively, which were the more effective treatments in the same order.

The acquired results generally show that administered therapies, particularly T6 and T7, play a protective defensive function as resistance-inducers against detrimental effects by improving anatomical performance and metabolic defense system. The data indicated that, clearly indicate that applied treatments especially, control (T14), T6 and T7 play a defensive protective role as resistance -inducers against adverse effects by enhancing the anatomical performance and metabolically defense system had a positive effect on all studied anatomical characteristics of root (Ø of root, cortex thickness, Ø of V.C and length of xylem arch), for stem (Ø of stem, cortex thickness, Ø of V.C and V.B thickness) as well as leaves (spongy and palisade tissues thickness and V.B thickness). While the used treatments of T1 and T13 had a negative effect on most of the studied anatomical characteristics of root, stem and leaf of of okra plants compared with unstressed plants treatments. These decreases were more pronounced with *M.* *javanica* (T1), *F.* *solani* (T13), and *M.* *javanica* + *F.* *solani* (T5).

## Discussion

The biocontrol potential of *P.* *chrysogenum* and *Trichoderma* *spp*. against root knot nematodes and *Fusarium* spp. was further confirmed by both laboratory and pot trial results. *Trichoderma* spp. species have been reported to suppress *Fusarium* spp. and plant parasitic nematodes^25–27^ due to their ability to produce hydrolytic enzymes that break down chitin in fungal cell walls^28^ as well as antibiotics^29^. *Trichoderma* spp. can directly control root-knot nematodes by producing poisonous compounds that prevent their penetration and growth^30–32^. For instance, *T.* *viride* and *T.* *harzianum* reduced the population of *M.* *javanica* in okra^33,34^ and tomato plants^35–37^. Their destructive activities include enzymatic disruption of eggshells and larval cuticles, as well as physiological abnormalities resulting from the production of diffusible toxic metabolites^38,39^. Studies by Sankari Meena, et al.^40^ and Poveda, et al.^41^ reported that the use of *T.* *harzianum* resulted in improved plant development metrics and a reduction in the fungal-nematode disease complex. Additionally, *T.* *harzianum* has been found to induce plant resistance and promote plant growth^42^.

Nematotoxic properties of diverse fungi's fungal culture filtrates add a new level of phytonematode biocontrol. Fungal culture filtrate of *T.* *harzianum* has been shown to have nematotoxic effects^43^, *Alternaria* *alternata*, *A.* *flavus*, *P.* *chrysogenum*, *Rhizoctonia* *bataticola*, *T.* *viride* have been recorded by Kumar, et al.^44^.

By decreasing the pathogen's development by up to 60.4%, the culture filtrate from *T.* *harzianum* displays antifungal action against *F.* *oxysporum* and confers resistance in soybean against *F.* *oxysporum*^45^. Bean pathogen growth can be inhibited by *T.* *harzianum* culture filtrate. In *Phaseolus* *vulgaris* seeds, *Pythium* *ultimum* lessens disease symptoms brought on by this pathogen^46^. The chitinase activity of cell-free culture filtrates improved, and they demonstrated fungus growth inhibition against the pathogens *Dematophora* *necatrix*, *F.* *solani*, *F.* *oxysporum*, and *Pythium* *aphanidermatum*. The effect was concentration-dependent, with the maximum growth inhibition rate for all the pathogens tested occurring at a concentration of 25% of the filtrate^28^.

The disease severity (%) dramatically decreased after the application of fungal culture filtrates, and plant growth metrics enhanced. These outcomes are consistent with those attained by Hegazy, et al.^47^ in sesame plants and Herrera-Téllez, et al.^48^ in tomato plants. Also, inhibition of mycelial growth by cultural filtrates of various bioagents^49^.

Microorganisms can produce certain antimicrobial compounds^50^. There are several opportunities for fungi, especially endophytic fungi, to create secondary metabolites, which are important sources of antimicrobial substances with growth-inhibitory effects on phytopathogens and can be employed as biological control agents^51,52^.

Obviously, results indicate the effect of different applied treatments: T1 [Nematode infection (*M.* *javanica*)], T5 [Nematode infection (*M.* *javanica*) + Fungus infection (*F.* *solani*)], T6 [Nematode infection (*M.* *javanica*) + Fungus infection (*F.* *solani*) + Overflowed with fungal culture filtrate (*P.* *chrysogenum*)], T7 [Nematode infection (*M.* *javanica*) + Fungus infection (*F.* *solani*) + Overflowed with fungal culture filtrate (*Trichoderma* spp.)], T13 [Fungus infection (*F.* *solani*)] on different anatomical features of okra root, stem and leaves compared with untreated plants (T14) which were evaluated on certain histological features of main okra root, stem and leaf at 60 days after planting. The fungal culture filtrate treatments led to an increase in the number of xylem vessels (NXV) in the vascular bundle. This rise in NXV seemed to be linked to the plant's ability to resist the fusarium wilt disease.Furthermore, the application of fungal culture filtrate resulted in an increase in the quantity and width of the fiber layers, as well as the thickness of the cambium region (Ø) in the root and vascular cylinder. This increase was observed in all the analyzed tissues, except for the cortex layers thickness. In addition, the Ø of the stem and epidermis, mesophyll tissue, and the thickness of vascular bundles (V.B.) in leaves were found to be important defenses against FOC infection, and were positively impacted in plants treated with fungal culture filtrate. Observations in plants treated with fungal culture filtrate revealed the presence of a fresh and regenerated vascular bundle. Pre-treating okra seeds with this filtrate led to beneficial changes in their water-conductive elements, facilitating greater absorption of water by the plants and reducing the spread of wilt disease. In fact, the tracheary components of the xylem, which function as the plant's water-conducting system, play a crucial role in supporting plant growth and development^53^. These results are consistent with the study of Ahmed and his colleagues on cucumber plants^54^. Internal root system design can be influenced by a variety of biotic and abiotic stimuli, including bacteria that encourage plant development (PGPM)^55^; the predominant genera of PGPM are *Rhizobia,* *Acetobacter,* *Bradyrhizobium,* *Bacillus,* *Pseudomonas,* *Trichoderma*^56^, and *Penicillium*^57^. Plant Growth-Promoting Microorganisms (PGPM) mainly induce changes in the architecture of the internal root system and the structure of root tissues by interfering with the plant's phytohormonal balance pathways that regulate cell origination and differentiation^58^. Several studies have reported that PGPM can provide protection to plants against phytopathogens by inducing the plant's defense mechanisms, which are known as induced systemic resistance (ISR). This phenomenon has been discussed in detail in the review article^59^. As a result of ISR, the cell wall is strengthened by increased lignin production and callose apposition, which slows the spread of phytopathogens across plant tissues^60^. As a result of PGPM-induced changes in root architecture and tissue structure, plants are able to absorb water and nutrients more efficiently, leading to faster overall plant development. Moreover, inoculation with *P.* *chrysogenum* and *Trichoderma* spp. can promote plant growth, enhance biomass accumulation, and improve soil nutrient uptake in plants^59^**.** A possible defensive mechanism against the okra Fusarium wilt has been suggested by the treatments' increased xylem vessel count and vascular bundle breadth.

Our research results are consistent with those reported by El-Sayed^61^,who demonstrated that using Trichoderma spp. culture filtrates to treat Faba bean plants against wilt pathogens produced strong evidence of resistance in anatomical examinations. When bioinducers are applied to plants through spraying, histopathological changes occur simultaneously with the elicitation of systemic acquired resistance, which is likely to enhance plant growth compared to unsprayed plants.

Overall, the data obtained clearly show that using fungal filtrates, particularly using fungal filtrate of *Penicillium* as a spray on plants (T6) or overflowed (T8), play a protective defensive function as resistance-inducers against detrimental effects by improving anatomical performance and metabolic defense system. It was obvious that control plants (nematode infection or fungus infection or both together) were negatively internally affected, which might be due to they not having developed mechanisms by which they protected against biotic stresses.

## Conclusion

The present study has shown that the bio-control agents gave good control of the disease complex in okra. The application of [Nematode infection (*M.* *javanica*) + Fungus infection (*F.* *solani*) + Overflowed with fungal culture filtrate (*P.* *chrysogenum*)] and [Nematode infection (*M.* *javanica*) + Fungus infection (*F.* *solani*) + spray with fungal culture filtrate (*P.* *chrysogenum*)] were significantly decreased the reproductive factors in the greenhouse and had the largest impact on nematode galling indices on okra roots. [Nematode infection (*M.* *javanica*) + Fungus infection (*F.* *solani*) + Overflowed with fungal culture filtrate (*P.* *chrysogenum*)] was the most effective medication in this regard, reducing illness severity by (28%) comparatively. On the other hand, the treatment [(Fungus infection (*F.* *solani*) + (Dovex 50% fungicide with irrigation water)] had the lowest relative illness severity documented (8%) at that time. The findings demonstrated that all examined anatomical properties of okra root, stem, and leaves were lowered by nematode infection, fungus infection, or nematode infection + fungus infection. Therefore, future studies should be focused on the development of economical and efficient mass production of these bio-agents to be used for the management of this disease complex under Egyptian conditions.

## Data Availability

All data available within the article.
